# A Comparison of Bevacizumab Plus TAS-102 and TAS-102 Monotherapy for Metastatic Colorectal Cancer: A Systematic Review and Meta-Analysis

**DOI:** 10.3389/fonc.2021.690515

**Published:** 2021-11-18

**Authors:** Xiaochen Chen, Huafeng Qiu, Yunwang Chen, Mingxing Wang, Pengfei Zhu, Shuangyue Pan, Yaya Deng, Liu Yang, Zheling Chen

**Affiliations:** ^1^ Department of Medical Oncology, Zhejiang Provincial People’s Hospital, People’s Hospital of Hangzhou Medical College, Hangzhou, China; ^2^ The Second Clinic Medical College of Zhejiang Chinese Medical University, Hangzhou, China; ^3^ Department of Medical Oncology, The Qingdao University Medical College, Qingdao, China; ^4^ Graduate School of Clinical Medicine, Bengbu Medical College, Bengbu, China

**Keywords:** TAS-102, bevacizumab, colorectal cancer, meta-analysis 3, survival

## Abstract

**Backgrounds:**

As a new oral chemotherapy drug, TAS-102 is currently recommended as the third-line treatment for metastatic colorectal cancer (mCRC). Recently, studies have reported the efficacy of TAS-102 combined with bevacizumab in colon cancer patients after standard treatment fails. Here, we evaluated the efficacy and safety of TAS-102 combined with bevacizumab versus TAS-102 as a single agent by a systematic review and a meta-analysis.

**Methods:**

PubMed, Web of Science and Cochrane libraries were searched. Studies involving bevacizumab combined with TAS-102 in mCRC were included. Study characteristics (author, year of publication, country et al.), efficacy (disease control rate(DCR), progression-free survival(PFS), overall survival(OS)) and adverse effects were extract from studies. Forest plots were created based on Cox model analysis.

**Results:**

After screening 550 studies, a total of 3 studies were included, which compared the safety and effectiveness of TAS-102 with or without bevacizumab. Analysis based on Cox regression showed that the combined treatment group had advantages in 6-month (OR= 2.93, 95% CI: 1.72 to 5.00, P<0.0001), 12-month(OR= 2.18, 95% CI: 1.24 to 3.81, P=0.006), and 18-month (OR=3.08, 95% CI: 1.34 to 7.12, P=0.008) OS. The combined treatment group demonstrated superiority in 6-month PFS rates (OR= 2.50, 95% CI: 1.18 to 5.31, P=0.02). The incidence of thrombocytopenia in the dual-drug treatment group was higher (OR= 1.96, 95% CI: 1.14 to 3.36 P=0.01). The proportion of serious adverse events were similar in tow groups (OR= 1.01, 95% CI: 0.76 to 1.34 P=0.93).

**Conclusion:**

Bevacizumab combined with TAS-102 could improve the prognosis of patients with mCRC who have failed standard treatment. In terms of side effects, the addition of bevacizumab did not increase serious adverse reactions, but the occurrence of thrombocytopenia was worth noting.

## Introduction

According to the Global Cancer Epidemiological Statistics (Globocan 2020) released by the International Agency for Research on Cancer (IARC) of the World Health Organization (WHO), the number of new cases and deaths of colorectal cancer in 2020 is 1.931,600 and 935,200 worldwide, ranking the third and second in all malignant tumors respectively (1). The most common metastatic routes of colorectal cancer are hematogenous metastasis, lymphatic metastasis, and implantation metastasis; the most common sites of metastasis are lymph nodes, liver, lung, and peritoneum (2). About 20% of patients with colorectal cancer are advanced at the time of initial diagnosis. An additional 25% of patients also developed distant metastases later in their diagnosis, although they were initially diagnosed as localized disease (3). Metastatic colorectal cancer (mCRC)has a poor prognosis, with a 5-year survival rate of less than 20%. Unresectable mCRC mainly relies on cytotoxic chemotherapy, biological therapies such as cell growth factor antibodies, immunotherapy, and systemic treatment with a combination of related treatments (4).

With the advancement of multidisciplinary treatment (MDT) models, the prognosis of patients with mCRC has been greatly improved. The first-line and second-line treatment of patients with mCRC is mainly through chemotherapy to control the disease (5, 6). In oxaliplatin and irinotecan-based chemotherapy regimens, the addition of anti-angiogenic drugs and anti-Epidermal growth actor receptor(EGFR) antibodies (RAS wild-type)have further improved the disease control rate and long-term survival of advanced patients (7, 8). The guidelines recommend regorafenib (9), or trifluridine-tipiracil hydrochloride mixture(TAS102) (10) as the third-line treatment for colorectal cancer (11). Currently, RAS mutation status testing is routinely performed on mCRC to guide the clinical use of anti-angiogenic drugs and anti-EGFR antibodies (RAS wild-type).

TAS-102 is an oral anti-tumor drug, including trifluorouridine (FTD, thymidine nucleic acid analog) and dipiphorin hydrochloride. On September 22, 2015, the U.S. FDA approved TAS-102 for the treatment of previously received chemotherapy based on fluoropyrimidines, oxaliplatin and irinotecan, as well as received/unsuitable for anti-vascular endothelial growth factor (VEGF) therapy, anti-EGFR therapy (RAS Wild-type) patients with mCRC (10, 12). A recent C-TASK FORCE study suggested that TAS-102 combined with bevacizumab has a disease control rate (DCR) of 64% in patients with refractory cancer who have failed other treatments (13). Subsequently, BiTS study optimized the dosage and interval of administration. The data showed that the biweekly TAS-102 plus bevacizumab dosing regimen is safe, and the median progression-free survival (PFS) and overall survival(OS)were 4.29 months and 10.86 months, respectively. The DCR was 59.1% (14).This study is a meta-analysis comparing the efficacy and safety of TAS-102 combined with bevacizumab and TAS-102 single-agent in patients with mCRC who have failed with standard treatment.

## Methods

### Systematic Literature Search

Search for published research in PubMed, Web of Science and Cochrane databases before March 25, 2021. Search keywords include “colorectal cancer”, “TAS-102”, “bevacizumab”, “metastasis”, and “clinical study”. After removing duplicates, two authors (Yang Liu and Chen Zheling) independently screened the title and abstract of the article. Then, we read the full text to further evaluate the inclusiveness of the literature. In addition, we also manually screened the reference list of the included studies to find other studies that may meet the screening criteria. The current meta analysis process was conducted according to the Preferred Reporting Items for Systematic Reviews and Meta-Analyses (15).

### Eligibility and Exclusion Criteria

All included literatures are studies comparing the efficacy of bevacizumab combined with TAS-102 and TAS-102 monotherapy in patients with mCRC. The inclusion criteria are as follows: (1) The study subjects are patients with colorectal cancer who have failed advanced standard treatment. (2) Double-arm clinical study, with comparable controls. (3) The study endpoint contains at least one efficacy data. The exclusion criteria are as follows: (1) Single-arm study. (2) The efficacy data missing. (3) The article type is review or other language types other than English.

### Evaluation of Methodological Quality

In this study, Newcastle-Ottawa Scale (NOS) and Jaded score are used for methodological quality evaluation (16). For the included non-randomized controlled studies, the NOS scale is used for evaluation. The evaluation criteria are divided into three parts (population selection, comparability and exposure evaluation). NOS uses a star system, with a full score of 9 stars, and studies with less than 6 stars are considered low quality. For the included randomized controlled studies (RCTs), the Jaded scale is used for evaluation. The evaluation contents of the Jaded scale include four aspects: randomization, concealment of allocation, double blinding, withdrawals and dropouts. The scale uses a score of 0-7, 1-3 is divided into low quality, 4-7 is divided into high quality.

### Data Extraction

We extract relevant data from the included studies for systematic analysis. The extracted data include the following: (1) Study characteristics (author, year of publication, country, study design, sample size). (2) Efficacy (DCR, PFS, OS). (3) Adverse reactions (grade III-IV adverse events, the incidence of specific adverse events).

### Statistical Analysis

We use Review Manager 5.3 software (The Cochrane Collaboration, Oxford, UK) for data integration and meta-analysis. The results of the meta-analysis are displayed in a forest diagram. The odds ratio (OR) and its 95% confidence interval (CI) based on frequency events and the hazard ratio (HR) and its 95% CI based on Cox regression were pooled for reporting. We used the Mantel-Haenszel (M-H) method to compare the clinical efficacy and toxicity of bevacizumab combined with TAS-102 group and TAS-102 single-agent group. The forest plot uses a vertical invalid line (the abscissa scale is 0 or 1) as the center, and multiple line segments parallel to the abscissa describe the effect size and CI of each included study, and a prism is used to describe the effect size and confidence interval of multiple studies combined. The heterogeneity of the included studies is judged by the I^2^ value. If there is no significant heterogeneity (I^2^<50% or I^2^ = 50%), use the fixed effects model (FEM); and in the case of considerable heterogeneity (I^2^>50%), the random effects model (REM) is used. OR and 95% CI are used to express statistical results. P <0.05 was considered statistically significant.

## Results

### Search Process and Results

**Figure 1:**
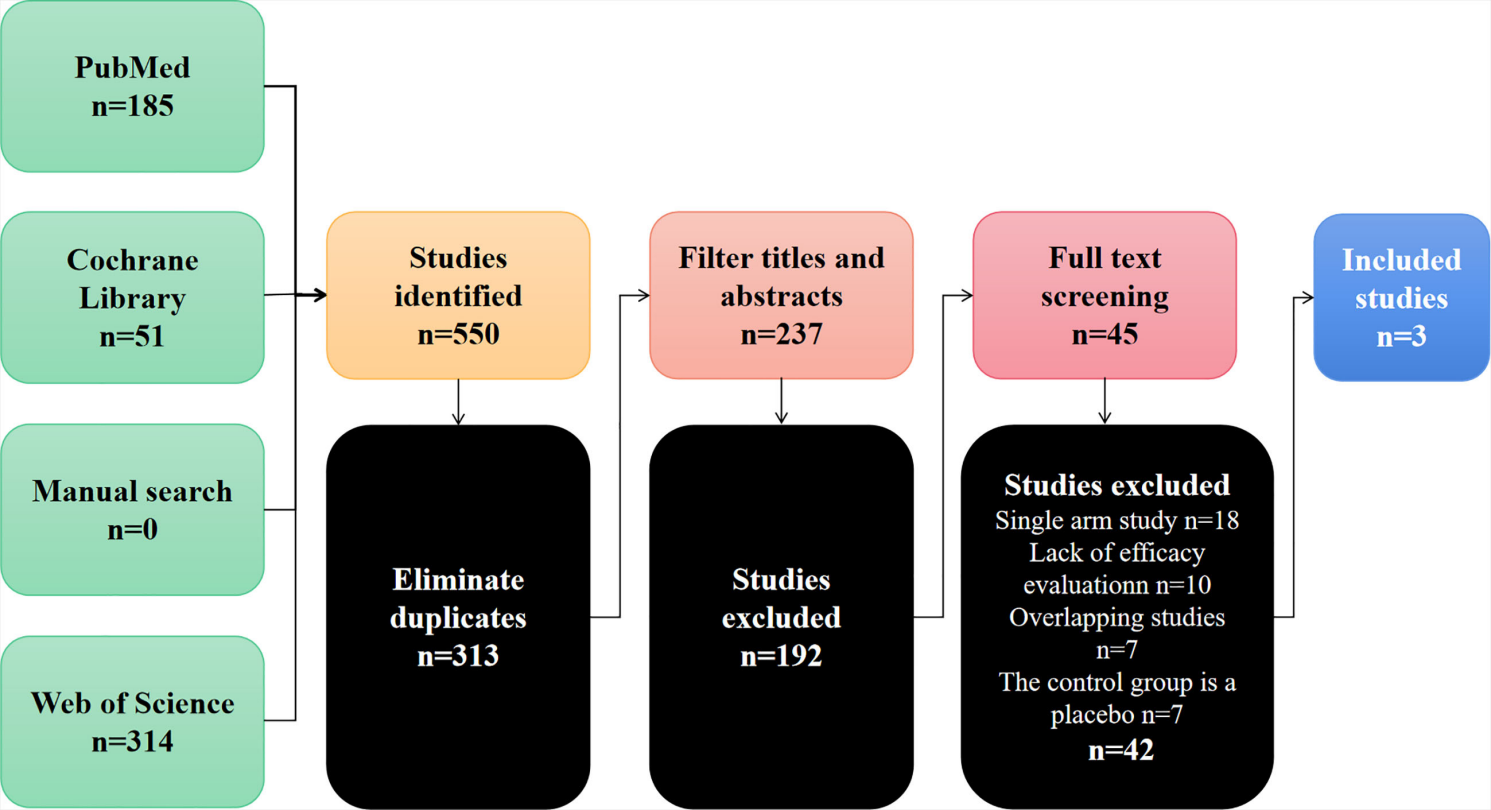
Flow graph of selection of included studies.

Through the preliminary screening of Pubmed, Cochrane Library, Web of Science and manual searches, 550 studies were identified. **Figure 1** summarizes the search flow chart of this study. After we eliminated duplicate documents, we carefully screened the titles and abstracts, and further eliminated the inconsistent studies. Finally, we further screened the remaining 42 studies by reading the full text. In the end, we eliminated those single-arm studies, studies with missing efficacy evaluation data, and studies with control groups that did not meet the requirements. A total of 3 studies were included in this meta-analysis (17–19). These studies compared the efficacy and safety of bevacizumab combined with TAS-102 versus TAS-102 as a single agent in mCRC. **Table 1** summarizes the basic characteristics of the included studies.

**Table 1:** Published articles reporting onTAS-102 monotherapy or combined with bevacizumab for the treatment of metastatic colorectal cancer.

Author	Year (study period)	Country	Study design	Procedure/Cases	
				TAS-102 monotherapy	TAS-102 plus bevacizumab
Hironori Fujii et al. (19)	2020 (2014-2018)	Japan	Retrospective, S	64	61
Daisuke Kotani et al. (17)	2019 (2014-2018)	Japan	Retrospective, S	66	60
Per Pfeiffer et al. (18)	2020 (2017-2018)	Denmark	RCT, M	47	46

RCT, randomized controlled trial; M, multicenter; S, single-center.

### Literature Quality Analysis

Among the 3 cohort studies included, 2 were single-center retrospective studies (17, 19), and once was a multi-center, prospective, phase II randomized controlled trial (RCT) (18). For retrospective studies, we use the NOS to assess methodological quality. For the RCT, we use the modified Jadad scale to evaluate. The results showed acceptable quality (NOS score > 6, Jaded score >3) for all of the included studies. The details of the assessment are shown in **Table S1** and **Table S2**.

### Efficacy

In this study, OS, PFS, and DCR data were extracted from the included clinical trials. The DCR refers to the proportion of cases with remission and stable disease after treatment. The data of PFS in one of the studies were missing, and only the data of two studies were included in the meta-evaluation of PFS.

**Figure 2:**
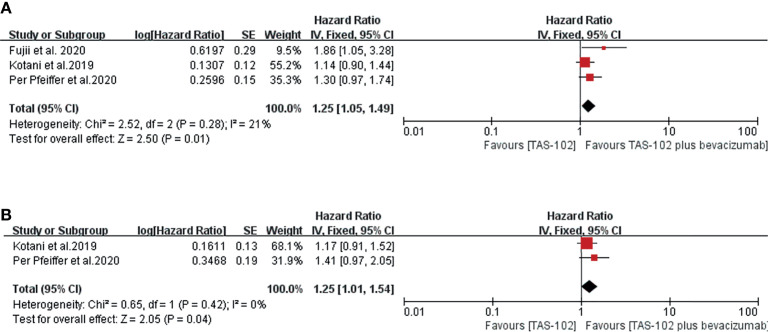
Forest plot of the meta-analysis comparing TAS-102 plus bevacizumab and TAS-102 in mCRC patients in terms of survival outcomes based on the Cox hazard model. **(A**) Overall survival. **(B)** Progress free survival. Horizontal lines represent 95% confidence intervals (CIs). df, degrees of freedom.

In terms of the OS and PFS results based on Cox regression, bevacizumab combined with TAS-102 showed benefit in both OS(HR=1.25; 95% CI: 1.05to1.49, P=0.01) and PFS (HR=1.25; 95% CI: 1.01 to 1.54, P=0.04) (**Figure 2**).

**Figure 3:**
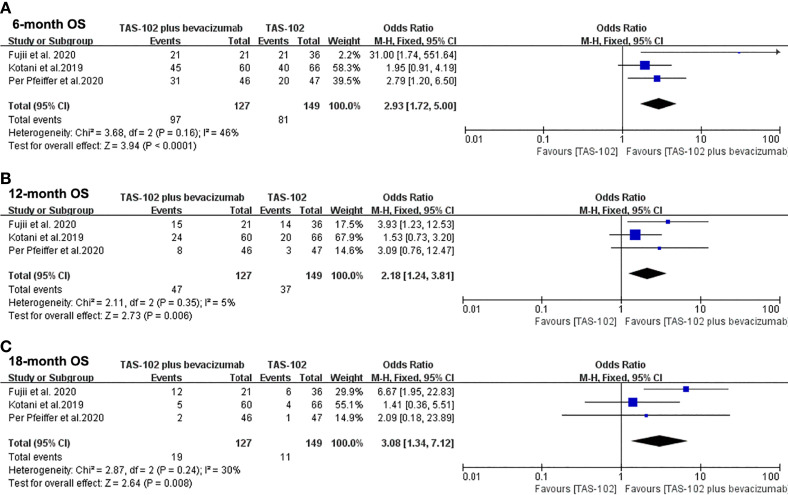
Forest plot of pooled relative risk for overall survival from included studies. **(A)** 6-month overall survival. **(B)** 12-month overall survival. **(C)** 18-month overall survival. Horizontal lines represent 95% confidence intervals (CIs). M-H, Mantel-Haenszel; df, degrees of freedom; OS, overall survival.

**Figure 3** showed the meta-analysis forest diagram of OS. The 6-month, 12-month, and 18-month OS data of the three studies were extracted. Bevacizumab combined with TAS-102 showed benefit in 6-month (OR= 2.93, 95% CI: 1.72 to 5.00, P<0.0001), 12-month (OR= 2.18, 95% CI: 1.24 to 3.81, P=0.006), and 18-month (OR= 3.08, 95% CI: 1.34 to 7.12, P=0.008) OS compared with TAS-102 monotherapy group.

**Figure 4:**
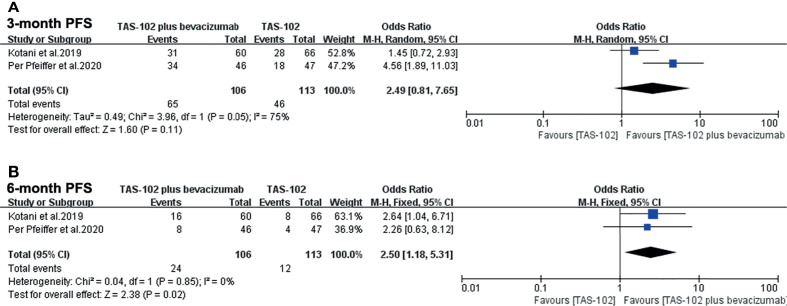
Forest plot of pooled relative risk for progress free survival from included studies. **(A)** 3-month progress free survival. **(B)** 6-month progress free survival. Horizontal lines represent 95% confidence intervals (CIs). M-H, Mantel-Haenszel; df, degrees of freedom; DFS, disease-free survival.

In the analysis of PFS in this meta-analysis, the 3-month PFS rates were not significantly different between two groups (OR= 2.49, 95% CI: 0.81 to 7.65, P=0.11) (**Figure 4A**). Bevacizumab combined with TAS-102 demonstrated superiority in 6-month PFS rates when compared with TAS-102 group(OR= 2.50, 95% CI: 1.18 to 5.31, P=0.02) (**Figure 4B**).

**Figure 5:**
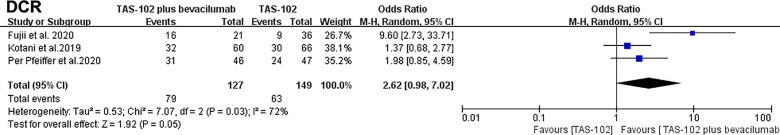
Forest plot of pooled relative risk for disease control rates from included studies. Horizontal lines represent 95% confidence intervals (CIs). M-H, Mantel-Haenszel; df, degrees of freedom; DCR, disease control rate.

For the comparison of DCR, there was no significant statistical difference in the analysis results between the bevacizumab combined with TAS-102 group and the monotherapy group(OR= 2.62, 95% CI: 0.98 to 7.02, P=0.05). It is worth noting that the P value between the two groups was 0.05 (**Figure 5**).

The analysis summary of the efficacy can be seen in part of **Table 2**.

**Table 2:** Summary of the results of the meta-analysis.

Result	Study number	Sample size (T+B/T)	Heterogeneity (*P*, I^2^)	Model	OR (95% CI)	*P*
**Clinical efficacy**						
DCR	3	127/149	72%	RE	2.62 (0.98,7.02)	0.05
6 months-OS	3	127/149	46%	FE	2.93 (1.72, 5.00)	<0.0001
12 months-OS	3	127/149	5%	FE	2.18 (1.24, 3.81)	0.006
18 months-OS	3	127/149	30%	FE	3.08 (1.34, 7.12)	0.008
3 months-PFS	2	106/113	75%	RE	2.49 (0.81,7.65)	0.11
6 months-PFS	2	106/113	0%	FE	2.50 (1.18, 5.31)	0.02
**AEs rates**						
AE(grade≥3)	3	588/511	0%	FE	1.01 (0.76, 1.34)	0.93
Neutropenia	3	127/149	62%	RE	1.88 (0.76, 4.63)	0.17
Anemia	3	127/149	0%	FE	0.79 (0.44, 1.43)	0.44
Thrombocytopenia	3	127/149	23%	FE	0.25 (1.14, 3.36)	0.01
Nausea	3	127/149	0%	FE	0.87 (0.43, 1.30)	0.30
Diarrhea	3	127/149	0%	FE	1.13 (0.60, 2.41)	0.71
Vomiting	3	127/149	40%	FE	1.51 (0.77, 2.97)	0.23
Fatigue	3	127/149	0%	FE	1.33 (0.78, 2.27)	0.29

T, TAS-102;B, bevacizumab; FE, fixed effects model; RE, random effects model; OR, odds ratio; DCR, disease control rate; AEs, adverse effects; OS, overall survival; PFS, progression Free Survival.

### Adverse Events

**Figure 6:**
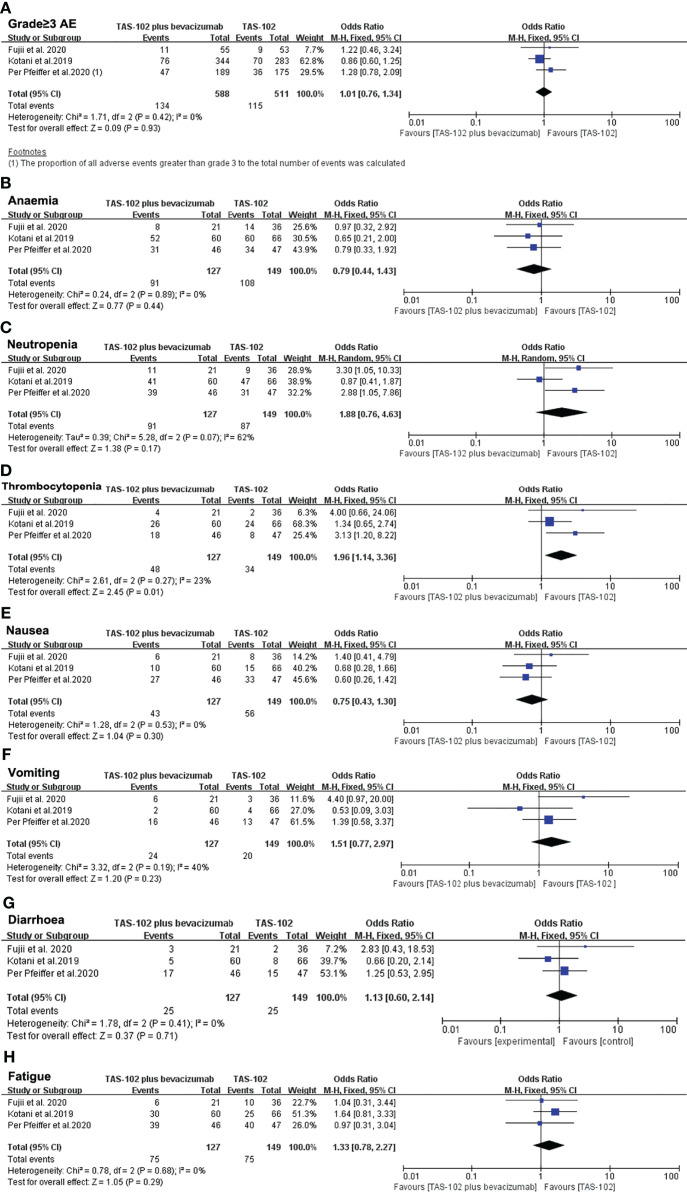
Pooled analysis for adverse effects. **(A)** The incidence of sever AEs (grade≥ 3), **(B–H)** represent the different side effects. Horizontal lines represent 95% confidence intervals (CIs). M-H, Mantel-Haenszel; df, degrees of freedom; AE, adverse effect.

For the analysis of AEs, we firstly focused on the incidence of sever AEs (grade≥ 3). All three studies reported the number of sever AEs. We included a total of 1,099 AEs, of which 249 (22.7%) were grade 3 or greater. The proportion of sever AEs did not increase significantly in TAS-102 combined with bevacizumab group compared with TAS-102 single agent (OR= 1.01, 95% CI: 0.76 to 1.34 P=0.93) (**Figure 6A**).

A subgroup analysis of hematologic toxicity profile showed that the incidence rates of anemia and neutropenia were not statistically different between the two groups (**Figures 6B, C**). However, the incidence of thrombocytopenia in the dual-drug treatment group was higher than that of single-drug treatment(OR= 1.96, 95% CI: 1.14 to 3.36 P=0.01) (**Figure 6D**). In terms of gastrointestinal reactions, there was no significant difference in the incidence of nausea (OR= 0.75, 95% CI: 0.43 to 1.30 P=0.30), vomiting (OR= 1.51, 95% CI: 0.77 to 2.97 P=0.23) or diarrhea (OR= 1.13, 95% CI: 0.60 to 2.14 P=0.71) between the TAS-102 combined bevacizumab treatment group and the TAS-102 monotherapy group (**Figures 6E**–**G**). There was also no significant difference in the incidence of fatigue between the two groups (**Figure 6H**).

The analysis summary of the AEs can be seen in part of **Table 2**.

### Risk of Publication Bias

For the results of treatment efficacy, we selected the study involving 6-month overall survival to draw a funnel chart to detect publication bias (**Supplementary Figure S1A**). And for adverse events data, we selected studies involving severe side effects (grade≥ 3) to draw a funnel chart (**Supplementary Figure S1B**). The funnel chart results showed that the images were symmetrical, indicating that there was no obvious publication bias in the current study.

## Discussion

The present study is the first meta-analysis of bevacizumab combined with TAS-102. The results suggested that bevacizumab combined with TAS-102 could bring survival benefits in patients with mCRC who have failed standard chemotherapy. Compared with TAS-102 single agent, combination therapy could prolong patient’s OS, improved PFS and DCR. In addition, combination therapy did not increase the incidence of serious AEs. The incidence of thrombocytopenia was higher than that of the monotherapy group. The results of this study were summarized in **Table 2**.

TAS-102 is a new, oral combination of trifluridine and tipiracil (1:0.5). Thymidine kinase 1 phosphorylates trifluridine, which acts as a substrate for DNA synthesis to interfere with DNA function. Tipiracil acts to inhibit thymidine phosphorylase to maintain the plasma concentration of trifluridine. A randomized, placebo-controlled phase II Japanese study confirmed that TAS-102 significantly prolonged the survival time of mCRC. The median overall survival of patients treated with TAS-102 was 9 months, compared with only 6.6 months in the placebo group (20). The follow-up phase III RECOURSE study verified the results of the above study. The disease control rate of the TAS-102 group was significantly higher (44% vs 16%), the survival time was longer (7.1 months vs 5.3 months), and the risk of death was reduced by 32% (10). According to the results of the RECOURSE study, TAS-102 has been marketed in the United States, Europe and Japan, and has been recommended by relevant guidelines (21, 22).

Folkman first proposed the theory that tumor growth and metastasis are dependent on new blood vessels in 1971 (23). Tumor angiogenesis depends on the breakage of the dynamic balance between angiogenic factors and angiogenic inhibitors, which is uncontrolled and immature, and eventually forms a distorted vascular network system (24, 25). Based on the theory of anti-tumor angiogenesis, researchers have focused on how to inhibit proangiogenic factors to effectively destroy tumor angiogenesis. In 2004, encouraging results were obtained in a large randomized controlled clinical trial of bevacizumab in metastatic colon cancer, showing that the treatment regimen of bevacizumab in combination with chemotherapy significantly improved prognosis (7). Since then, more and more new drugs that inhibit the binding of vascular endothelial growth factor (VEGF) and VEGF receptor have entered clinical studies. Preclinical studies have shown that during the treatment of bevacizumab, various angiogenic related factors, the non-dependent axis of VEGF and the role of tumor-associated macrophages (TAM) in the tumor micro-environment are compensatively up-regulated, and the replacement pathway is activated, resulting in accelerated growth of drug-resistant clones and increased invasion of tumor cells (26, 27). Therefore, there is an urgent need to introduce the next generation of antiangiogenic drugs. Continuous antiangiogenesis can make the tumor dormant and help to control the malignant progression of the tumor. Therefore, antiangiogenesis therapy should be continued (28, 29).

At present, in the entire management of mCRC, the first-line and second-line treatments are mainly targeted drugs combined with standard chemotherapy, and the third-line treatment recommends regorafenib, fruquintinib(in China) or TAS-102 as a single agent. There is a network meta-analysis to compare the three drugs of third-line treatment, and there is no significant difference in efficacy (30). However, unlike the small molecule TKI inhibitors, TAS-102 is essentially a cytotoxic drug. The C-TASK FORCE study is the first study to evaluate the safety and therapeutic activity of TAS-102 combined with bevacizumab as a salvage treatment. The median PFS by central assessment was 3.7 months and 5.6 months by investigator assessment. The results showed that the combination of the two could be safely used in salvage treatment and was a promising anti-tumor treatment. The results of the study met the study endpoint, confirming that TAS-102 combined with bevacizumab treatment can improve 16-week PFS, which exceeded the preset threshold in the phase 2 study of TAS102 single-agent. Recent clinical data from Denmark showed that the median PFS ofTAS-102 combined with bevacizumab treatment reached 4.6 months, which was 2.6 months inTAS-102monotherapy group (P=0.0015) (13, 18). The concentration of trifluorothymidine in TAS-102 in plasma and tumor DNA increases in a dose-dependent manner. The higher the concentration is, the higher the anti-tumor activity will be presented. However, the increase in plasma concentration of TAS-102 will inevitably affect the treatment tolerance. In the C-TASK FORCE study, the plasma concentration of trifluorothymidine was close to the previous single-drug concentration, and bevacizumab increased the accumulation and phosphorylation of trifluorothymidine in the tumor. Preclinical studies have shown that the combination of TAS-102 and bevacizumab increases the concentration of phosphorylated trifluorothymidine in tumor cells. Therefore, the combination of the two only increases the concentration of trifluorothymidine in tumor DNA without increasing systemic exposure, thereby increasing anti-tumor activity and prolonging survival (31). Our meta-analysis included three studies comparing the TAS-102 plus bevacizumab with TAS-102 single agent (17–19). Comprehensive data analysis objectively suggests that dual-drug therapy can bring benefits to mCRC patients who have failed standard treatments.

Can bevacizumab combined with TAS-102 become a first-line or second-line treatment? The recommended first-line treatment for metastatic colorectal cancer is a two- or three-drug regimen consisting of oxaliplatin, irinotecan, or fluorouracil, combined with targeted drugs (bevacizumab, cetuximab, and panitumumab). However, there are clinically some patients who cannot tolerate intensive treatment. Such patients usually recommend fluorouracil therapy with or without bevacizumab (32). The SOLSTICE (NCT03869892),a randomized phase III trial being conducted in the European Union, andTASCO-1,a randomized phase II trial, are studies randomly compared TAS-102 plus bevacizumab and capecitabine plus bevacizumab for first-line treatment of unresectable metastatic colorectal cancer that cannot be intensively treated (33, 34). Results from TASCO1showed the PFS of TAS-102 plus bevacizumab and capecitabine plus bevacizumab were 9.23 and 7.8 months, and the HR was 0.71; the OS was 22.3 and 17.7 months, and the HR was 0.78 (34). The safety analysis also suggested that TAS-102plus bevacizumab treatment was well tolerated. Therefore, TAS-102plus bevacizumab shows promising clinical activity and acceptable safety in first-line unresectable metastatic colorectal cancer patients who are not suitable for intensive treatment. In TRUSTY trial, non-inferiority of TAS-102 plus bevacizumab to standard of care (35). Regarding the subgroup analysis, some patients in the control group used the S-1 regimen, and the HR of these patients reached 2.57 (1.39-4.44), which significantly elongated the OS of the control group.S-1 and TAS-102 are both modified fluorouracil preparations. In this study, the addition of S-1 in the control group may have affected the study endpoint. In addition, some KRAS wild-type patients in the experimental group were not exposed to anti-EGFR antibodies during the first and second-line treatment stages, which may lead to a shortened overall OS in the experimental group. All in all, TAS-102combined with bevacizumab did not show non-inferiority compared to standard chemotherapy in second-line treatment. The follow-up subgroup analysis is still in progress. It is hoped that people who benefit from the combination of TAS-102 and bevacizumab can be found, and there are no new safety issues in the second-line application. Recent data from the KSCC1602 study showed that bevacizumab combined with TAS-102 was an effective and well-tolerated regimen for elderly patients with untreated mCRC over 70 years of age. The median PFS was 9.4 months, the median OS was 22.4 months, and the ORR was 40.5% (36). This means that this treatment plan is expected to become one of the standard treatment options for metastatic colorectal cancer. Due to its good safety, it can be called the preferred treatment plan for elderly patients. SUNLIGHT study (NCT04737187) is designed as a randomized phase III comparison study evaluating the efficacy and safety of TAS-102 in combination with bevacizumab versus TAS-102 monotherapy inpatients with metastatic colorectal cancer who are refractoly or intolerant to standard treatment. The research is still in progress and the results are worth expecting from clinicians.

Our results showed that in most of the comparison of AEs, the addition of bevacizumab did not increase the incidence rates of sever AEs. However, the results showed that the incidence of thrombocytopenia was higher in bevacizumab plus TAS-102 group. Bevacizumab is an anti-vascular drug, and the occurrence of hematological toxicity is considered to be rare. However, there are still some cases reported that bevacizumab can cause severe immune-mediated thrombocytopenia (37–39). When using bevacizumab combined with TAS-102, the side effects may still be superimposed. The C-TASK FORCE study showed that the frequency of neutropenia increased in the TAS-102 combined with bevacizumab group (13). It has been reported that VEGF blockade increases the risk of neutropenia when combined with chemotherapy. In the C-TASK FORCE study, only one patient was hospitalized for neutropenic fever and recovered well. Therefore, these side effects can be controlled, but G-CSF supportive treatment is needed, or the dose needs to be temporarily reduced or stopped, which is the same as when TAS-102 is used as a single agent (RECOURSE) (10). In fact, the median treatment interruption time and relative dose intensity of the C-TASK FORCE study are consistent with the previous TAS-102 single-agent application.

This study also has some limitations. Due to the screening conditions of clinical studies, this study only included three large randomized clinical studies, and the selection of patients was biased. There are also many clinical research data that are not publicly available, so they cannot be fully included. Therefore, more random prospective study data are still needed to truly clarify the value of TAS-102 combined with bevacizumab in the treatment of advanced colon cancer.

## Conclusion

This meta-analysis suggested that bevacizumab combined with TAS-102 could improve the prognosis of patients with mCRC who have failed standard treatment. Bevacizumab combined with TAS-102 compared with TAS-102 single drug could be better in terms of OS, PFS and DCR. In terms of side effects, the addition of bevacizumab did not increase serious AEs, but the occurrence of thrombocytopenia was worth noting.

## Data Availability Statement

The original contributions presented in the study are included in the article/**Supplementary Material**. Further inquiries can be directed to the corresponding authors.

## Author Contributions

ZC and LY gave contributions to conception and design. ZC, YC, and MW reviewed the literature and designed the article structure. ZC, PZ, HQ, and YD contributed to the acquisition and analysis of data. ZC, XC, and SP gave interpretation of data. ZC and XC were major contributors in writing the manuscript. LY revised and edited the manuscript critically for important intellectual content. All authors contributed to the article and approved the submitted version.

## Funding

This work is supported by the National Natural Science Foundation of China (No. 81802623).

## Conflict of Interest

The authors declare that the research was conducted in the absence of any commercial or financial relationships that could be construed as a potential conflict of interest.

## Publisher’s Note

All claims expressed in this article are solely those of the authors and do not necessarily represent those of their affiliated organizations, or those of the publisher, the editors and the reviewers. Any product that may be evaluated in this article, or claim that may be made by its manufacturer, is not guaranteed or endorsed by the publisher.

## Supplementary Material

Supplementary Figure S1 Funnel plot for publication bias. **(A)** Funnel plot in the studies involving 6-month overall survival; **(B)** Funnel plot in the studies involving severe side effects (grade≥ 3).
