# Supplementary figures and images for: A Comparison of Bevacizumab Plus TAS-102 and TAS-102 Monotherapy for Metastatic Colorectal Cancer: A Systematic Review and Meta-Analysis
Source: Front Oncol. 2021 Nov 18;11:690515. doi: 10.3389/fonc.2021.690515 (PMC8637322; doi:10.3389/fonc.2021.690515)

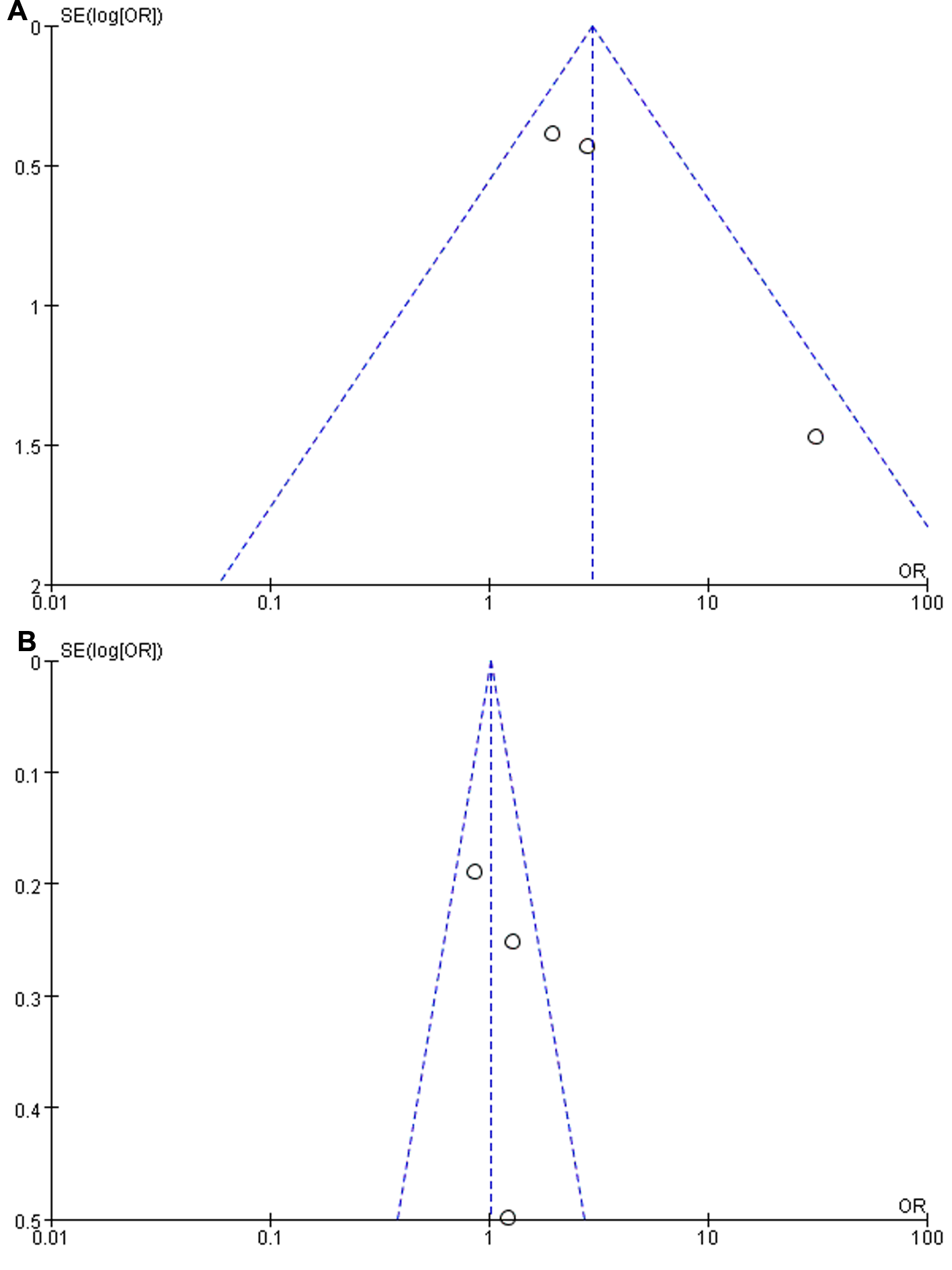

Supplement: Supplementary Figure S1 — Funnel plot for publication bias. (A) Funnel plot in the studies involving 6-month overall survival; (B) Funnel plot in the studies involving severe side effects (grade≥ 3). [file Image_1.tif]
