# Supplementary material for: A Comparison of Bevacizumab Plus TAS-102 and TAS-102 Monotherapy for Metastatic Colorectal Cancer: A Systematic Review and Meta-Analysis
Source: Front Oncol. 2021 Nov 18;11:690515. doi: 10.3389/fonc.2021.690515 (PMC8637322; doi:10.3389/fonc.2021.690515)
Supplement: Supplementary file 2 [file Table_1.docx]

**Table S1** Methodological assessment of the included retrospective studies

| **Author** | **Selection** | | | | **Comparability** | **Outcome** | | | **Quality scores** |
| --- | --- | --- | --- | --- | --- | --- | --- | --- | --- |
|  | **(1)** | **(2)** | **(3)** | **(4)** |  | **(5)** | **(6)** | **(7)** |  |
| HIRONORI FUJII et al. | 1 | 1 | 1 | 1 | 2 | 1 | 0 | 1 | 8 |
| Daisuke Kotani et al. | 1 | 1 | 1 | 1 | 2 | 0 | 1 | 1 | 8 |

1. Representativeness of the exposed cohort; (2) Selection of the non-exposed cohort; (3) Ascertainment of exposure to implants; (4) Outcome of interest was not present at start of study; (5) Assessment of outcome; (6) Was follow up long enough for outcomes to occur; (7) Adequacy of follow up of cohorts.

**Table S2** Methodological assessment of the included randomized controlled trial (Jadad score)

| **Author** | **Randomization** | **Concealment of allocation** | **Double blinding** | **Withdrawals and dropouts** | **Jadad scores^a^** |
| --- | --- | --- | --- | --- | --- |
| Per Pfeiffer et al. | 1 | 2 | 2 | 1 | 6 |

^a^Methodological quality of meditative movements studies reviewed using Jadad scoring criteria.Ttotal score is 7. Score 1 to 3 considered as low quality; score 4 to 7 considerd as high quality.
